# Potentiation Physiologically Proven

**Published:** 1893-11

**Authors:** Gustav Jaeger

**Affiliations:** Stuttgart, Germany


### POTENTIATION PHYSIOLOGICALLY PROVEN.

PROF. DR. GUSTAV JAEGER, STUTTGART, GERMANY.

[Translated by B. Fincke, M. D. Continued from page 488.]

### VI.—DEDUCTIONS AND CONSIDERATIONS.

At the close of the foregoing chapter the *subjective* consequences have been drawn from chapter V, but the matter must still be discussed particularly.

1. Toward the *objective* side I would like to render the following points conspicuous:

(*a.*) Whoever proves medicines only on the *sick*, does not realize what he is doing, for when he sees effects he cannot judge whether the medicine itself and directly has called out the symptoms, or whether they are the sequel of the liberation of the disease-matter, and even if he can do that, he does not know at all, whether the liberation of the disease-matter was owing to his medicamentous interference or whether it is only a *post hoc.* If, however, we can with certainty each time call out a phenomenon on the healthy which he does not show before and after the cessation of the action, then we know quite distinctly that it was the medicine which had acted. This gives an infallible objective insight into the action of the medicine. (See, also sub. 1 below.)

(*b.*) It would be a very naïve conception of the healing action of medicines if one would believe that they act in the body only by their *presence* upon the disease like a bug-bear or a scare-crow—that from sheer terror of the simile or ·ison it takes to flight. The medicine, if it is to act, must make its appearance *active, moving.* For this purpose it has exclusively two powers at its command, the *chemical affinity* or the *physical motor-energy;* a third rational and plausible does not exist.

(*c.*) It is doubtless that there are medicine-substances which act by their *chemical affinity,* and I know such from quite exact and extensive proving on myself and others. There is, *e. g.*, the group of the *odorants,* which destroy high atomic organic substances under the pressure of Oxygen and change them into low atomic ones. The most powerful in this relation are *Camphor* and the mixture of ethereal substances and Acetic Ether called *Ozogen* used as atmospheric purifier. But here the *chemical action* is in direct ratio to the *mass* and decreases with it. If, therefore, we want to have this chemical action, we must *not* attenuate, and if it is done, nevertheless, it is no potentiation of the action, but a weakening. The *chemical action follows the allopathic principle: much helps much and little little.*

(*d.*) On the other hand, there is another group of substances, with *pronounced healing actions* of which nobody will maintain that they act chemically, for the reason that they do not decompose themselves. To this class belongs as a type the *Sulphuric Ether* at present so much employed as injection by the allopaths, then *Moschus*, formerly so much used as a resuscitating remedy, in general a whole series of substances the most striking common property of which is their *volatility* (champagne, all alcoholics, in short, all so-called restoratives, belong to this class). They produce their healing action only by their motor-energy, their *volatility*.

(*e.*) In homœopathic circles it is frequently maintained that the efficacy of homœopathic medicines depends upon their *fine distribution*, the large superficial development. A perfectly indistinct conception lies at the bottom of this assertion. It is only correct as long as we have to deal with *solid visible* parts as with a powder, but as soon as the substance is *dissolved* in a fluid, it is decomposed in its *last* constituent parts, the *molecules*, hence all the *surfaces* which it possesses are perfectly liberated and developed, and no power of the world can do anything more, except the molecules are exploded into their atoms, but then the substance as such has disappeared. Therefore, if the efficacy of the medicine-substances consisted in this, the potentiation of the solutions had not only no sense, but the contrary would have to ensue: decrease of action, since with the attenuation the number of the molecules, therefore, the sum of the efficacious surface decreases. Hence this theory of potentiation is nonsense.

(*f.*) Others have the conception that the power of the *succussions* communicates itself to the medicine-substance. This is *a priori* impossible. First of all indeed these succussions are not without efficacy, but the effect is merely a *heating* of the shaken fluid. This, heat, however, simply escapes again, because it is conductive heat (Leitwärme) if nothing is done to prevent it, and this is the case when simultaneously with the succussion an attenuation of a substance, dissolved in it, is connected. In order to prove this, I have carefully made the same succussions with the same Alcohol without simultaneous attenuation as in potentiation, and proved these succussed fluids neuro-analytically; when I waited till the heating had again disappeared, the neuro-analytical effect never surpassed the limit of error. The reversed experiment, viz., the *attenuation without succussions* and its result is already described on page 30 of my "Neural-Analyse," this is the counter-proof. If the *attenuation* is made without *succussion*, a change occurs similar to potentiation; only such an *unshaken* potency corresponds in relation to its animating effect only to one, perhaps half as high (an unshaken 30th potency at that time corresponded to a shaken 15th potency). This, however, proves only, that without shaking the potentiation is imperfect, because the layer of fluid adhering to the walls of the vial from the foregoing potency does not mix so rapidly with the newly added Alcohol that in the next pouring out it is gone—*i. e.*, perfectly mixed.

(*g.*) Dr. Goullon has somewhere said that the potencies of *Jenichen* shaken with his athletic arms have acted stronger because he could shake them stronger. If this fact is true, I judge about it differently on the ground of my rich experience with *Anthropine;* the anthropine of an athlete possesses the potency of an athlete and the medicine-potencies which such a man prepares, contain always his anthropine and to the action of the potentiated medicine associates itself the action of Athleto-anthropine. Therefore, the matter is this: Whoever denies that with the so-called potentiation an increase of power of the medicinal action intended by Homœopathy takes place, we have here nothing to do, but he who admits it will be unable to hold fast to any other explanation than that which I have given, for the others so far known to me, do not stand the test of experiment.

(*h.*) Let us take the matter up once more from the beginning. I have said: a medicine can act only in two ways: either by its chemical affinity or by the motor-energy of its molecules. If, now, substances like granite, gold, kitchen salt, etc., which are chemically quite indifferent, and otherwise possess no volatility *per se*, shall be made into a medicine, motor-energy must be imparted to it, otherwise they are physiological, and, with it, therapeutical nothings. Now, it is quite true that with the transference of a substance in a fluid or dissolved form, a motion of its molecules begins, and it can now be physiologically efficacious (*corpora non agunt nisi soluta*), but the *measure of its action* stands on two points: (*a.*) On its specific motor-rhythm and its proportion to that of the substances of the body; (*b.*) On the intensity of this motion. Of these two things, the substance has the first *per se*, and nothing is to be done or changed in that respect. But it is clear, if we succeed to induce a change of the second, the intended medicine-action must increase in energy and velocity. Now, *Hahnemann* has perceived that this is done by the *attenuation method*, and all have found the same who proved the matter earnestly. Experience teaches the same in regard to the refining of the alcoholic beverages by storage, and there is no clearer proof of it than the involuntary spasms which finally appear when sufficiently sensible persons operate with sufficiently high attenations. To this must be added, what I want here to remark merely as an appendix: on using highest potencies, sensible persons experience a remarkable excitement, which is not only felt by the persons themselves—an utterance which I frequently heard of others, and which corresponds also entirely with my sensation, is, "it is as if I would like to strike everything to pieces"—but also the people around perceive it in the change of behavior of the prover in his heightened irritability. This excitement often lasts the whole day.

(*i.*) We now must dedicate a special consideration to the twitchings in different directions. Factors which liberate vital motions are called *vital stimuli.* The general law is that, with increasing strength of the vital stimuli, their effects increase, and that twitchings, and even cramps, belong to the effects which are obtained by vital stimuli only at a higher irritability. Therefore, if I change a vital stimulus, which in original form produces no twitchings, in such a manner that it produces them, and that it finally calls out cramps of almost all the muscles, I have augmented the strength of its irritation, and this is done also when the production of these maximal effects does not succeed with all individuals. Hence, the potentiation—*i. e.*, the attenuation of a substance—is an augmentation of its irritating strength, and in a medicine it is an increase of the medicine-action. Nobody will contend, I trust, that a medicine cannot act otherwise than physiologically, and if I am able to increase its *physiological action upon the healthy,* I gain with it also an *increased action* upon the sick.

(*k.*) The fact that high potencies can produce twitchings and spasms calls forth its comparison with *electricity,* which produces this phenomenon in especially high degree. That *electricity* is a *healing factor,* Homœopathy does not dispute, and this capability it owes to its power of liberating motion. If, now, high potencies possess also this capability, it is, as such, quite independent of it, a healing factor like electricity whether it be a *simile* or not. It is, however, a better one, and for this reason: Electricity acts fully only upon the *conducting* tissues (muscle and nerve); the badly conducting tissues it can influence much less, but the high potency does not depend upon the *conduction,* but upon the *diffusion* which carries it to the badly-conducting tissues as easily as to the well-conducting, it therefore *acts more universally.*

When a homœopathic physician gives all his medicines in high potency, therefore, in a form in which *every* medicine-substance has similar, even universally-acting motorial properties as Electricity, he acts with every substance whether it is the *simile* or not, as it were electrically, therefore also therapeutically. On the contrary, he who operates with low potencies, which do not have this general electrical action independent of the quality of the substance, has accomplished nothing if he has not hit upon the *simile.* Or will anybody maintain that in the numerous medicine-substances which I have proved on myself, and from every one of which I experienced the spasmodic actions, the concerning one has always been my *simile?*

In the first place, on the whole, I was not sick; secondly, in the comparative measurement of the neutral salts, I have not measured them in alternation, but I have each time on one day measured about 6–7 *different* substances in the same potency, hence, they all together could not have been my *Similia.* If the word "Electro-Homœopathy" were not abused, for the label of secret remedies, and therefore, made impossible for scientific investigations, I would like to apply it to the Homœopathy which works with high potencies, because this *always*—even if it misses the *simile*—acts similarly upon the sick as with an electric current, or, to make another comparison, as with a *volatile animating remedy*. Moreover, I do not want to be misunderstood, I do not want to be known as falling into the opposite error of neglecting the Law of the Similars. What I say, is that *both* fundamental laws are of equal correctness and *equal* importance, and that the use of the low potencies is a fundamental fault which could not have been made, if either one had been perfectly clear on the point what properties a substance must have in order to act physiologically and therapeutically, or if one had applied correspondingly the proving on the healthy upon potentiation, or if they had held fast what *Hahnemann* had taught about potentiation. The use of the low potencies is an apostasy from Hahnemann.

(*l.*) Toward another side these medicine-cramps afford clearness. I have already said (sub. *a*) why we cannot obtain clearness in experimenting on the sick, this must be taken up again. The twitchings high potencies can produce in the healthy cease on inhalation already within the *first* minute after cessation of the inhalation. If the medicine had been swallowed, they last somewhat longer, as seen in Table I, but never even as long as 15 minutes. This shows clearly that the *power*, or better, the velocity of motion which the molecules of the substance have obtained by attenuating, soon decreases after the ingestion, and an equilibrium succeeds in which the medicine acts no more as a stimulus. From this it appears: when striking processes are observed on the *sick* and on the healthy hours and days after taking the medicine, these stand to the medicine-action only in an *indirect* relation. The medicine can do nothing else with its "potency" than call forth in the tissue a kind of shock with a shortly lasting molecular *concussion* (which, of course, it will do best and most certain, if in the tissue resides its *ison* or *simile*). When this, its main mission is evidently accomplished; it takes leave, and what may happen afterward, is only the *consequence* of this concussion of the tissues. If this is sufficient, phenomena will appear which, on the sick, we have to call *crises,* healing crises. But these are directly nothing else than poisoning symptoms, called forth by the *disease poisons,* set free in consequence of the decumulation (Entspeicherung). It seems to me, however, as if with many homœopathic physicians the idea had taken firm hold that these were after-actions which had to be placed to the account of a remainder of medicine-substance in the body, as if this in a measure once more revived and aroused itself to an after-action. I have formerly often heard and read such utterances, but in vain tried to obtain a conception of it how that could be possible. Now here I have obtained full light, the concussion by the medicine can lead immediately to a crisis, but it can, also, in many ways tarry, because the properly expelling factor is the vital energy of the tissue. If this by the first shock has gained a small surplus, it will indeed turn the scale in its favor, but for an extensive success, even amounting to an actual healing crisis the gained surplus must have a distinct magnitude, which is only gained *step by step,* and can be gained when the first success is utilized. The assertion of many high potentialists that a single dose of medicine could be sufficient to induce an after-action, lasting through months with final healing, contains, therefore, for me throughout nothing improbable; the more powerful the first concussion had been the more certain that first turning point is reached which enables the physician to leave the process to itself, or, in better words, to the vital energy of the tissues of the patients, which increases with every shock.

(2.) Now in conclusion, something on the *demonstrative* side of the medicine-cramps of the high potencies.

(*a.*) Here I call to mind the excitement which the words of the Prussian Cultusminister produced, when in the House of Representatives he pointed out that Homœopathy could not prove the efficaciousness of its remedies as allopathy; thereupon, everybody called for hospitals in order to furnish the proof. I say, the proof can and shall never be furnished because it can never be proved whether it was the medicine which healed the patient. The allopath can demonstrate on the healthy to everybody the efficaciousness of his medicines, for if he gives him an emetic he vomits, if a laxative he produces diarrhœa, if chloral sleep, and if antipyrin perspiration. To these palpable proofs of the medicine-action on the healthy on the *allopathic* side, the *homœopath*, who works with low potencies, can oppose nothing. The high potentialist on the contrary.can take up the contest on the healthy by the fact that he can produce upon him twitchings, and even actual spasms. This is an inestimable argument.

"You would be right in this respect if the highest potencies would always and on everybody produce at least twitchings. But this is not the case as you say yourself."

When an allopath makes this objection to me I ask him quietly whether he could maintain that—*e. g.*, one and the same emetic in one and the same dose in *all* individuals produces vomiting; Chloral, with all of them, sleep; Opium, with all of them, constipation, etc.? He will admit that this is not the case. Well, what is sauce for the goose is sauce for the gander. The reader knows the joke about swallowing the whole homœopathic pocket-case. I have not proved it myself, but know that if it can be done at all, this would be only allowable with low potencies. The late Professor v. Rapp has told me that he tortured such a braggard with high potencies so much that he struck sail and declared himself vanquished.

Homœopathy complains of the oppressed situation in our country. It cannot complain if it allows the weapons to rust idly which it possesses in order to conquer a position, and such a weapon is the high potency, and if, instead of using it, it tries to insinuate itself into the favor of the ruling school with low potencies. He who does not swing the hammer is and remains the anvil. There is no third in life.

(*b*.) They complain that it is so difficult among young medical men to enlist adherence to Homœopathy in order to secure a sufficient increase of regularly educated physicians. Here is a case in point: A young homœopathic physician who, in the beginning, found it very hard to play the eccentric and apostate among his comrades, and to put himself in the little enviable position of a heretic and rebel, studied Homœopathy at an office where they thought potentiation might be neglected. When the young physician was not satisfied with his success, he made several times the following experiment: when he had a case in which a favorable termination could with certainty be assured, owing to the state of strength, he gave one time the *Simile* indicated according to the books, the other time a substance the indication of which was as much as possible the contrary, as inappropriate as possible, and lo! the cure followed in the last case just as promptly and completely as after the careful selection of the *Simile*, and of course would have ensued if nothing had been given. Is it a wonder that this young man all at once mistrusted the whole of Homœopathy? As long as the homœopaths of Germany believe in diminishing the contrast between them and the allopaths, and increasing the adherence to Homœopathy among physcians by sacrificing the principle of potentiation, the effect will be the *direct contrary*. Only by means of high potencies the physiological action of which can be proved on the healthy, and which on the sick give lightning-like success, it can make propaganda successfully among the medical younger generation. Critical heads cannot be conquered by low potencies because they offer nothing palpable.

"Very well! But I must remind you again that the actions of the high potencies not always and not in all persons appear palpable—*i. e.*, as twitchings, and that just for this reason, we cannot convince everybody."

It is not at all necessary, on the contrary: Homœopathy requires physicians who are constituted so finely that they know how to calculate finenesses and how to use them. The allopaths may keep those who cannot do that, and the homœopaths may be saved from such physicians, who are good for nothing than for mechanics (surgeons). By this I will, however, not have said that they have passed amendment: if they lay aside their skepticism, if with earnestness they will lay their hands on, then with the practice also their physical impotence will disappear, as I have already formerly pointed out. On the dry wood indeed we shall not live to see much, but we expect something from the green.

